# Supplementary material for: Multiomics Studies on the Effects of High-Temperature Stress on Male Sterility in Gossypium barbadense
Source: Int J Mol Sci. 2025 Apr 14;26(8):3693. doi: 10.3390/ijms26083693 (PMC12027491; doi:10.3390/ijms26083693)
Supplement: Supplementary file 1 [file ijms-26-03693-s001.zip › ijms-3548101-Supplementary Material.pdf]

Multiomics studies on the effects of high-temperature stress on male sterility in *Gossypium barbadense*

Jiangbo Li [1,†], Xiaojuan Deng[1], Man Gao[1], Tao Lv[1], Yongsheng Cai[1], Yanying Qu[1], Quanjia Chen[1], Kai Zheng[1,*]

Affiliations

[1]Engineering Research Centre of Cotton, Ministry of Education/College of Agriculture, Xinjiang Agricultural University, 311 Nongda East Road, Urumqi, 830052, China

Jiangbo Li , Xiaojuan Deng , Man Gao , Tao Lv , Yongsheng Cai , Yanying Qu, Quanjia Chen , Kai Zheng

Corresponding author

Correspondence to Kai Zheng.

Highlights

·The changes in hormones and related core genes of *Gossypium barbadense* under high-temperature stress were investigated through combined transcriptomic and metabolomic analyses.

·We successfully constructed a hormone coexpression network and a genome-wide coexpression network related to anther cracking under high-temperature stress.

· There are two genes that may play a crucial role in the male sterility of sea Island cotton: one is discussed in terms of function (ALA4) showing potential influence, and the other is discussed in terms of structure (SBP1) showing significant role.

Abstract

High-temperature (HT) stress has been recognized as one of the main factors r estricting the normal growth and development of cotton and severely affects fib er quality and yield. To elucidate the regulatory mechanism of male sterility-rel

ated hormones in *Gossypium barbadense* under HT stress, we explored candidate genes closely related to male sterility in *G. barbadense*. We studied the expression profiles of hormones and genes in the anthers of *G. barbadense* GB150 under HT stress by combining transcriptomic and metabolomic analyses. Through combined analysis of the transcriptional metabolism of GB150 anthers of *G. barbadense*, we determined the contents of ABA, JA, SA, IAA, tZR and GA_{20} and the expression of genes related to biosynthetic pathways and signal transduction pathways. The results revealed that the ABA and JA contents significantly increased after HT, the IAA, tZR and GA_{20} contents significantly decreased, and the SA content did not significantly change after HT. We then used weighted gene coexpression network analysis (WGCNA) to further analyze the interactions among hormones, transcription factors, and core genes and constructed hormone coexpression networks and genome-wide coexpression networks. Through these network analyses, we ultimately identified 10 candidate genes closely related to male sterility in *G. barbadense*. Using qRT–PCR, resequencing data from 221 *G. barbadense* materials revealed that ALA4 (*Arabidopsis thaliana* has been proven to be associated with male fertility) and SBP1 (two stop gains in the gene structure) may play important roles in the process of male sterility in *G. barbadense*. The results of this study provide a theoretical basis for the molecular mechanism of male sterility in *G. barbadense*.

Keywords: *Gossypium barbadense*, transcriptome, metabolome, anther, male sterility

Abbreviations

HT: High-temperature

G. Barbadense: Gossypium barbadense

qRT-PCR: Quantitative real-time polymerase chain reaction

DEGs: Differentially expressed genes

PCA: Principal components analysis

WGCNA: weighted gene co-expression network analysis

ABA: Abscisic Acid

JA: Jasmonic Acid

SA: Salicylic Acid

GA_{20}: Gibberellic Acid 20

tZR: trans-Zeatin Riboside

IAA: 3-Indoleacetic acid

VED: Violaxanthin de-epoxidase

ABF: Abscisic Acid (ABA) Responsive Element Binding Factors

NCED: 9-cis-epoxycarotenoid dioxygenase gene

CYP: Cytochrome P450

AOS: Allene Oxide Synthase

LOX: Lysyl oxidase

MYC: Transcription factor MYC

JAR: Jasmonate Responsive

OPR: 12-oxo-phytodienoic acid reductase

NPR: Non-expressor of pathogenesis-related

PAL: Phenylalanine ammonia-lyase

ICS: Isochorismate synthase

UGT: UDP-glucuronosyltransferase

BSMT: Benzoic acid/salicylic acid carboxyl methyltransferase

TGA: TGACG motif binding factor

ARF: Auxin Response Factors

AUX: Auxin/Indole-3-Acetic Acid

SAUR: Small Auxin Up RNA

ARR: Arabidopsis response regulators

AHP: Histidine-containing Phosphotransfer Protein

ZOG: Zeatin O-glucosyltransferase

miaA: tRNA dimethylallyltransferase

CKX: Cytokinin Oxidase/Dehydrogenase

DELLA: DELLA protein

GA2OX: Gibberellin 2-oxidase

GID: Gibberellin receptor

PIF: Phytochrome-interacting Factors

Supplementary information

Additional files 1: Table S1.Anther dehiscence-related candidate genes.
Additional files 2: Fig. S1 GB150 different periods of heat resistance evaluation of the comprehensive value of coclustering.
Additional files 3: Fig. S2 Expression of eight TFs.
Additional files 4: Fig. S3 Temperature change chart.

Table S1 Anther dehiscence-related candidate genes

Top five ids of the blue module	gene_name	Gene Description	Expression trend
Gbar_A09G027130	ALA4	Probable phospholipid-transporting ATPase 4	down
Gbar_D04G003870	DUS3	tRNA-dihydrouridine(47) synthase [NAD(P)(+)]	down
Gbar_A01G019500	DTX42	Protein DETOXIFICATION 42	down
Gbar_A03G000700	NAC071	NAC domain-containing protein 71	down
Gbar_D13G025850	PI4KG1	Phosphatidylinositol 4-kinase gamma 1	down
Top five ids of the blue module	gene_name	Gene Description	Expression trend
Gbar_A08G001080	CAF1-11	Probable CCR4-associated factor 1 homolog 11	up
Gbar_A02G001830	SBP1	Selenium-binding protein 1	up
Gbar_A07G004670	APS1	Acid phosphatase 1	up
Gbar_D10G023850	UXS6	UDP-glucuronic acid decarboxylase 6	up
Gbar_A09G025410	BCE2	Lipoamide acyltransferase component of branched-chain alpha-keto acid dehydrogenase complex, mitochondrial	up

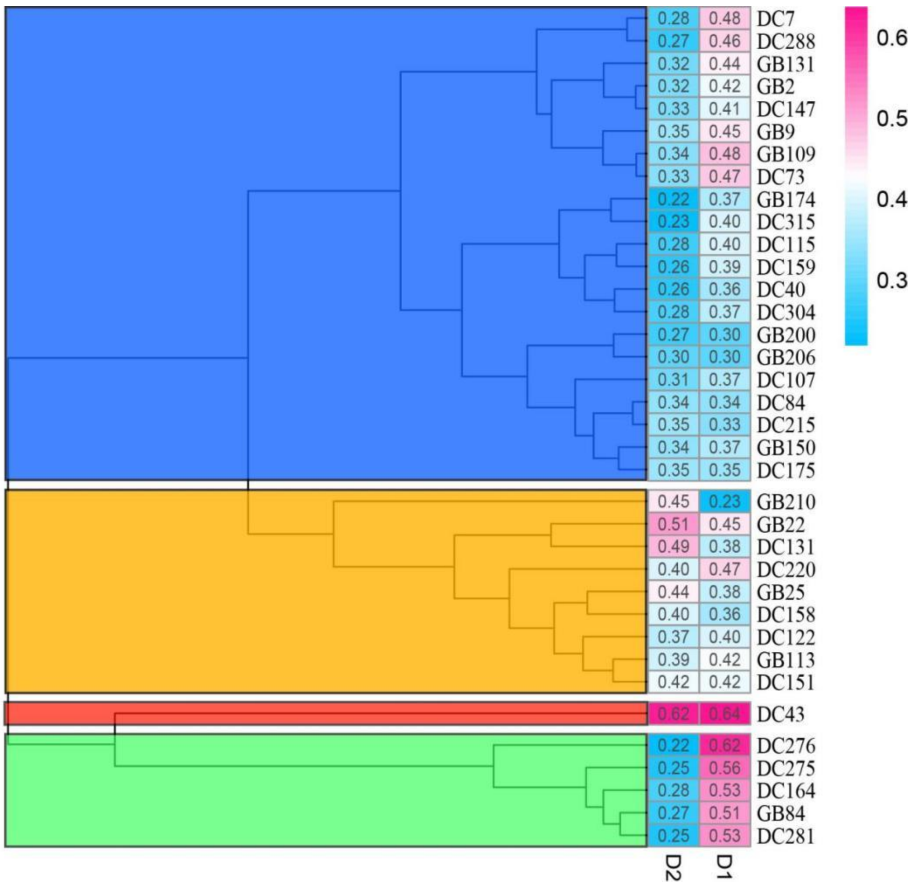

Fig. S1 GB150 different periods of heat resistance evaluation of the comprehensive value of coclustering. D1: 36 germplasms of extreme flower organs at the adult stage; D2: 36 germplasms of extreme flower organs at the seedling stage.

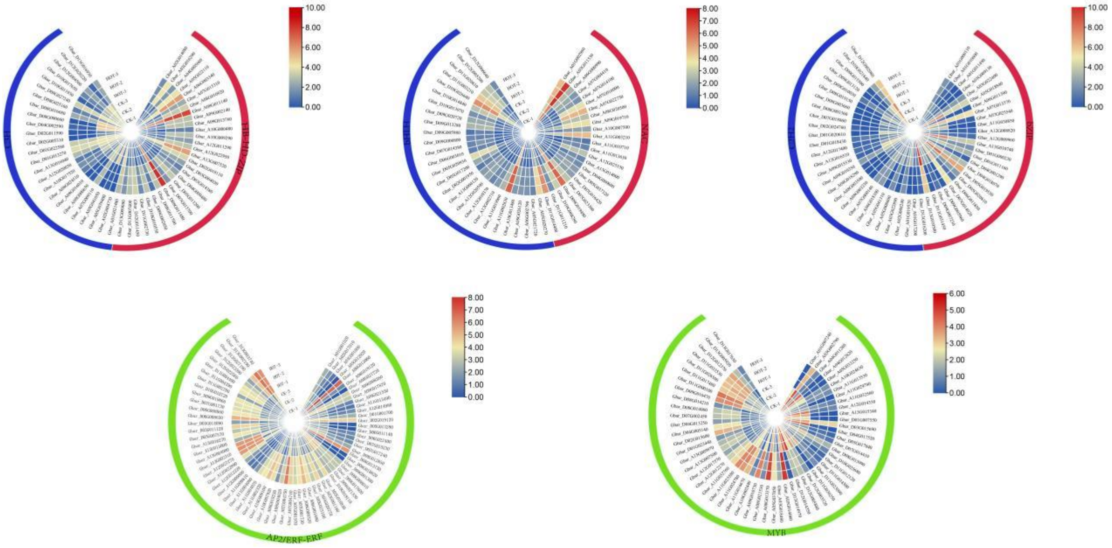

Fig. S2 Expression of eight TFs.

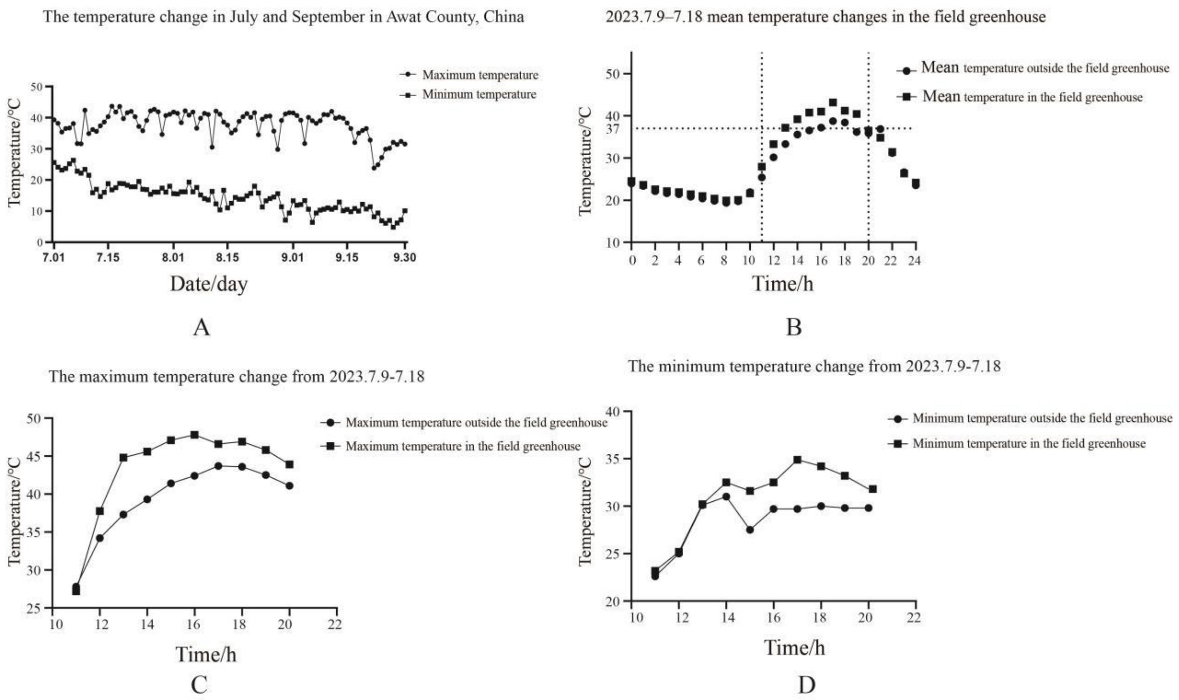

Fig. S3 Temperature change chart.(A) The temperature change in July and September in Awat County, China.**(B)**2023.7.9–7.18 mean temperature changes in the field greenhouse.**(C)**the maximum temperature change from 2023.7.9-7.18.**(D)** the minimum temperature change from 2023.7.9-7.18.
